# E'jiao and Cubilose Formula Induced Antioxidant Activity and Improved Collagen Expression of Human Skin Fibroblasts During Oxidation Damage

**DOI:** 10.1111/jocd.16604

**Published:** 2024-11-12

**Authors:** Hang Tie, Xiao Xie, Zichen Zhang, Jianling Zhang, Liang Xu, Haihua Ruan, Tao Wu, Hongyang Zhang

**Affiliations:** ^1^ Tianjin Key Laboratory on Technologies Enabling Development of Clinical Therapeutics and Diagnosis, School of Pharmacy Tianjin Medical University Tianjin People's Republic of China; ^2^ School of Pharmacy Tianjin Medical College Tianjin People's Republic of China; ^3^ Chinese Academy of Inspection & Quarantine Greater Bay Area Zhongshan Guangdong People's Republic of China; ^4^ National Engineering Research Center for Gelatin‐Based Traditional Chinese Medicine Liaocheng Shandong China; ^5^ Dong‐E E‐Jiao Co. Ltd Liaocheng Shandong China; ^6^ Tiangong University, Tianjin Medical College Tianjin People's Republic of China; ^7^ Tianjin Key Laboratory of Food Biotechnology, College of Biotechnology and Food Science Tianjin University of Commerce Tianjin China

**Keywords:** antioxidant activity, cubilose, E'jiao, oxidation damage, skin health

## Abstract

**Background:**

As traditional Chinese medicinal materials, E'jiao and cubilose are rich in various bioactive substances with good antioxidant, anti‐inflammatory, and immune‐regulating effects.

**Aim:**

To obtain the optimal ratio of synergistic effect between E'jiao and cubilose.

**Methods:**

The antioxidant capacity of E'jiao and cubilose digestive fluid was evaluated in vitro, as well as the intracellular oxidation balance between HSF cells and 3D whole‐skin model induced by H_2_O_2_.

**Results:**

E'jiao, cubilose, and their different ratios of composites had better scavenging ability against free radicals such as DPPH (2,2‐Diphenyl‐1‐picrylhydrazyl). Using HSF cells induced by H_2_O_2_ as an oxidative damage model, it was found that the combination of E'jiao and cubilose in a ratio of 2:3 significantly enhanced the activities of SOD, GSH‐PX, and CAT enzymes compared to the separate treatments of E'jiao and cubilose, as well as other combination ratios. It effectively reduced the accumulation of reactive oxygen species caused by H_2_O_2_ in HSF (Human Skin Fibroblast) cells and protected the integrity of the cells. Further analysis using flow cytometry and a 3D full‐thickness skin model revealed that the combination ratio of 2:3 increased the proportion of H_2_O_2_‐treated cells in the S%+G2% phase from 19.1% + 7.4%–22.1% + 28.8%, helping oxidatively damaged cells partially recover their proliferative capacity. It also promoted the expression of collagen I and collagen IV in the 3D full‐thickness skin model, with improvement rates reaching 168.00% and 123.68%, respectively.

**Conclusions:**

These findings indicate that the combination of E'jiao and cubilose in a ratio of 2:3 exhibits good synergistic effects, enhancing the ability of cells to resist oxidative damage, promoting cellular renewal and metabolism, and improving skin antiwrinkle capacity.

## 1 Introduction

Long‐wave ultraviolet (UVA) and medium‐wave ultraviolet (UVB) radiation from the sun can reach the Earth's surface [1, 2]. UVA radiation, which is the main component of solar ultraviolet radiation, can cause varying degrees of damage to the skin. It is particularly associated with skin inflammation, pigment deposition, acne, aging, and epidermal thickening [3, 4]among these, the most significant is the induction of oxidative damage to the skin. Oxidative damage can lead to the overproduction of reactive oxygen species (ROS) in skin cells, disrupting their antioxidant defense systems, causing lipid peroxidation reactions, affecting signaling pathways, and damaging cellular structures or functions. This can ultimately contribute to the development of photoaging, malignant tumors, and other related diseases [5]. Therefore, how to prevent skin photo‐oxidation or alleviate oxidative damage to the skin and delay skin aging has been of great concern.

Currently, there is ample research indicating that E'jiao and cubilose have certain beneficial effects on skin health [6, 7, 8, 9, 10, 11, 12]. E'jiao is a solid gel derived from boiling and concentrating the dried or fresh skin of donkeys, which are members of the horse family. It is abundant in beneficial components such as sugars, amino acids, and vitamins. E'jiao offers multiple effects, including blood nourishment, moisturization, antioxidant properties, anti‐inflammatory effects, and the ability to delay aging [13]. Based on the research by Dong et al., it has been found that E'jiao can enhance the activity of relevant antioxidant enzymes, reduce the levels of malondialdehyde (a product of oxidative damage), and inhibit the expression of aging‐related proteins in aging mice by increasing D‐galactose. These findings indicate that E'jiao has the potential to delay the aging process [6]; in addition, E'jiao digesta can reduce the depth of wrinkles and prevent skin aging caused by UV irradiation by inhibiting the decline in the expression of Type IV collagen and elastin proteins [7]. Cubilose, the saliva secretion of the swiftlet species belonging to the family Apodidae during its breeding season [14], contains abundant nutrients such as proteins, various amino acids, carbohydrates, inorganic elements, and fats [15]. It possesses multiple effects, including anti‐inflammatory, antioxidant, antiaging, skin whitening, and improvement of skin function [16]. In a study conducted by Wong et al., it was found that cubilose digestive fluid exhibits stronger whitening activity and osteogenic activity [10]. The study by Ghassem et al. demonstrated that the peptide components in simulated digestion products of cubilose exhibit high antioxidant capacity and can protect cells from oxidative damage induced by H_2_O_2_ [17]. Furthermore, the triglycerides found in cubilose are rich in unsaturated fatty acids, which can exert antioxidant effects [9, 18]. The sialic acid present in cubilose also exhibits skin whitening effects and can improve skin functionality [8].

While there have been numerous studies on the individual benefits of E'jiao and cubilose, the mechanisms and effects of their combined formulation on skin health have yet to be fully understood. To comprehensively assess the effectiveness of composite mixtures containing E'jiao and cubilose, We prepared simulated gastrointestinal digestive fluids for E'jiao, cubilose, and various ratios of their combined formulations, conducted in vitro antioxidant capacity evaluation of the digestive fluids, along with assessment of intracellular oxidative balance using H_2_O_2_‐induced HSF cells and a 3D full‐thickness skin model, aiming to determine the optimal combination ratio of E'jiao and cubilose in terms of influencing skin antioxidant and antiaging properties and provide reference for exploring their mechanisms and potential applications in maintaining skin health.

## 2 Materials and Methods

### 2.1 Preparation of E'jiao and Cubilose Samples

The self‐made samples used in this study include six groups, which consist of E'jiao and cubilose: E'jiao (E), cubilose (C), E:C mass ratio = 1:3, E:C = 2:3, E:C = 1:1, and E:C = 4:3.

### 2.2 Cell Culture

Human dermal fibroblasts, HSF cells, were cultured in Dulbecco's modified Eagle's medium (DMEM), supplemented with 10% (v/v) fetal bovine serum (FBS) and 1% (v/v) penicillin/streptomycin in a humidified atmosphere with 5%CO_2_ at 37°C. HSF from third to 14th passages were used in the experiments.

### 2.3 Preparation of Enzyme‐Digested E'jiao and Cubilose

E'jiao the cubilose thoroughly using a mortar and pestle, then mixed it with E'jiao according to the desired sample ratio. Dissolved the mixture in 9 mL of simulated gastric fluid (without enzymes, containing 0.3 M sodium chloride, pH = 2), and added 1 mL of artificial gastric fluid. The mixture was digested in a light‐avoiding manner for 2h at 37℃ on a shaker operating at 100r/min. After 1 h of gastric digestion, add 3 mL of artificial intestinal fluid to each 10 mL of the digested gastric fluid. Adjust the pH to 6.8 using a 0.4 M NaOH solution. Digested the mixture in a light‐avoiding manner for 2 h at 37°C on a shaker operating at 100 r/min. The resulting solution after digestion was ready for analysis [19, 20].

### 2.4 Free Radical Scavenging

#### 2.4.1 DPPH Radical Scavenging Assay

DPPH assay was conducted using the DPPH assay kit (Beyotime, Jiangsu, China) [21]. In each experiment, 80% methanol was used as a blank, and DPPH dissolved in 80% methanol was used as a control. The DPPH radical scavenging rate is expressed by the following equation:

$$\text{DPPH scavenging rate}\;\left(\%\right)=\left[\left(1-\left(A_{2}-A_{1}\right)÷A_{0}\right)\times 100\right]\%$$

where *A*_1_ represents the absorbance of the control group, *A*_2_ represents the absorbance of the sample group, and *A*_0_ represents the absorbance of the blank group.

#### 2.4.2 Superoxide Anion Radical (O_2_ ·^−^) Scavenging Assay

Superoxide anion radical (O_2_·^−^) scavenging assay was performed as shown by Liu et al. [22]. The absorbance *A*_0_ was measured before mixing with the sample solution.

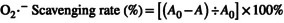

where *A*_0_ represents the absorbance of the control group and *A* represents the absorbance of the sample group.

#### 2.4.3 OH· Scavenging Assay

The ability of E'jiao and cubilose to inhibit OH·activity was determined using the Fenton method [23], with some modifications. Different samples of E'jiao, cubilose, and their complex compounds were sequentially mixed with a 9 mmol/L ferrous sulfate solution, sample solution, ultrapure water, and 0.024% hydrogen peroxide solution. The mixture was allowed to stand at room temperature for 10 min. Then, 9 mmol/L salicylic acid solution was added and mixed thoroughly, followed by incubation at 37°C for 30 min. The absorbance was measured at 510 nm. The ability of the sample to inhibit OH·activity is expressed by the following equation:

$$\mathrm{OH}\cdot \text{Scavenging rate}\;\left(\%\right)=\left[\left(A_{0}+A_{x0}-A_{x}\right)÷A_{0}\right]\times 100\%$$

where *A*_0_ represents the absorbance of the blank group, *A*_x_ represents the absorbance of the sample group, and *A*_x0_ represents the absorbance of the unexposed hydrogen peroxide.

### 2.5 Cell Viability Assay

Cells were seeded at 5 × 10^4^ cells per well into 96‐well plates and incubated with the indicated drugs. Subsequently, 90 μL of fresh medium was added to cells containing 10 μL of Cell Counting Kit‐8 (Beyotime, Jiangsu, China) solutions and incubated for 1 h in an incubator of 5% CO_2_ at 37°C. Absorbance at 450 nm was measured using a microplate reader.

### 2.6 Hydrogen Peroxide Treatment

Stable hydrogen peroxide stock solution was diluted with PBS and added to fresh culture medium. The H_2_O_2_ concentrations ranged from 0 to 400 μmol/L (0, 80, 90, 100, 200, 300, and 400 μmol/L) per well. HSF cells were exposed for 1 h.

### 2.7 ROS Assay

HSF cells (1 × 10^4^) were seeded in a 24‐well plate and treated with 100 μL of different samples and an equal volume of PBS (phosphate buffer saline). After that, they were treated with 100 μmol/L H_2_O_2_ solution for 1 h. Then, 200 μL of DCFH‐DA solution (dissolved in DMSO; final concentration of 10 μM) was added and incubated at 37°C for 15 min. The incubation solution of DCFH‐DA was removed, and cells were washed three times with serum‐free culture medium to remove unabsorbed DCFH‐DA. After washing, 100 μL of PBS solution was added to each well. The fluorescence of ROS was measured at an excitation/emission wavelength of 485/525 nm.

### 2.8 SOD, CAT, and GPX Activity Assay

HSF cells (1 × 10^4^) were seeded in a 24‐well plate and treated with 100 μL of different samples and an equal volume of PBS. After that, they were treated with 100 μmol/L H_2_O_2_ solution for 1 h. The activity of SOD, CAT, and GSH was measured following the instructions provided by the assay kit supplier (Beyotime, Jiangsu, China).

### 2.9 Cell Cycle Analysis of HSF Cells by Flow Cytometer

Cell cycle analysis was performed 48 h after cell treatment. Harvested cells were washed once with cold PBS. The washed cells were fixed in 70% ethanol and incubated at 4°C for 24 h. After fixation, cell clumps were removed using a cell strainer. The cells were then washed with PBS and stained with RNase (0.25 μg/mL) and PI (50 μg/mL) for analysis using a flow cytometer. Data analysis was conducted using FlowJo software.

### 2.10 Cell Membrane Permeability Assay

Cell membrane permeability analysis was performed 48 h after cell treatment. The supernatant was collected and cells were collected after two washes with PBS. The cells were then resuspended to create a cell suspension, which was mixed with the supernatant. The mixture was combined with a 1:1 ratio of 0.4% trypan blue‐staining solution, thoroughly mixed, and the number of stained and unstained cells was counted to calculate the cell viability rate.

### 2.11 3D Culture of Skin Equivalents

Preparation before testing: Transfer the model to a six‐well plate. Except for the blank control group (BC), the negative control group (NC), positive control group (PC), and sample group were subjected to UVA irradiation with a dose of 35 J/cm^2^. After irradiation, 5% (v/v) administration was added, and the six‐well plate was incubated in a CO_2_ incubator (37°C, 5% CO_2_) for 24 h. The model surface residues of the test substance were washed with sterile PBS solution, and any remaining liquid inside and outside the model was gently wiped away with a sterile cotton swab. Fluorescent staining: After incubating the probes, the model was taken out and placed in a 1.5‐mL EP tube for fixation with 4% paraformaldehyde. After 24 h of fixation, freeze embedding and sectioning are performed. Images were taken under a microscope and analyzed using Image‐ProPlus image processing software.

### 2.12 Statistical Analysis

The experimental data were statistically analyzed and plotted using SPSS. The results are presented as mean ± standard deviation (mean ± SD), with at least three replicates for each experimental group. The significance level was set at *p* < 0.05.

## 3 Results

### 3.1 Analysis of the Free Radical Scavenging Abilities of Different Digestive Fluids Including E'jiao, Cubilose, and Its Combination

The DPPH radical, superoxide anion radical, and hydroxyl radical are commonly used to evaluate the in vitro antioxidant capacity of products. The results of the clearance of these radicals by the digestive fluids of E'jiao, cubilose, and their complex are shown in Table 1. The digestive fluids of E'jiao, cubilose, and their complex all demonstrated antioxidant capacity. Compared to E'jiao, cubilose exhibited stronger clearance ability against DPPH radicals, reaching 75.05%. When E'jiao and cubilose were combined in a ratio of 1:1 and 4:3, their clearance rates were similar, at 71.66% and 71.52%, respectively. The highest DPPH radical clearance rate, reaching 76.00%, was observed when E'jiao and cubilose were combined in a ratio of 2:3, but with no significant difference from the individual digestive fluids of E'jiao and cubilose (*p* > 0.05). Compared to cubilose, E'jiao exhibited stronger clearance ability against superoxide anion radicals, reaching 79.54%. When E'jiao and cubilose were combined in a ratio of 1:1, the clearance ability of the complex against superoxide anion radicals was lower, at 66.63%. When the combination ratio of E'jiao and cubilose was 1:3 and 4:3, the clearance abilities against superoxide anion radicals were similar, at 74.57% and 73.13%, respectively, showing improvement compared to the 1:1 ratio. When the ratio was 2:3, the complex exhibited a clearance ability of 76.23% against superoxide anion radicals, but there was no significant difference compared to the individual digestive fluids of E'jiao and cubilose (*p* > 0.05). The clearance abilities of E'jiao and cubilose individual digestive fluids against hydroxyl radicals were similar, at 28.78% and 28.81%, respectively. When the combination ratio of E'jiao and cubilose was 1:3, the complex demonstrated the lowest clearance ability against hydroxyl radicals, at 21.82%. As the combination ratio changes, the clearance ability of the complex against hydroxyl radicals gradually improves. When the ratio was 1:1, the clearance rate was 22.14%. When the ratio was 4:3, the clearance rate was 23.31%. When the ratio was 2:3, the clearance ability against hydroxyl radicals was 25.42%. The complex exhibits lower clearance ability against hydroxyl radicals compared to the individual digestive fluids of E'jiao and cubilose.

**TABLE 1:** Summary of the clearance results of DPPH, superoxide anion, and hydroxyl radicals by different samples.

Sample	DPPH radical (%)	Superoxide anion radical clearance (%)	Hydroxyl radical clearance (%)
E	71.12 ± 3.62ab	79.54 ± 1.46a	28.78 ± 0.96a
C	75.05 ± 2.44a	73.79 ± 1.72b	28.81 ± 1.57a
E:C = 1:3	68.41 ± 2.33b	74.57 ± 1.96b	21.82 ± 0.93c
E:C = 1:1	71.66 ± 2.78ab	66.63 ± 1.94c	22.14 ± 0.63c
E:C = 4:3	71.52 ± 2.44ab	73.13 ± 1.84b	23.31 ± 1.22bc
E:C = 2:3	76.00 ± 2.34a	76.23 ± 2.32ab	25.42 ± 1.03b

*Note:* Values are presented as mean ± SD. Different letters indicate a significant difference with *p* < 0.05, as determined with one‐way ANOVA.

Abbreviations: C, cubilose; E, E'jiao; E:C = 1:1, the mass ratio of E'jiao to cubilose is 1:1; E:C = 1:3, the mass ratio of E'jiao to cubilose is 1:3; E:C = 2:3, the mass ratio of E'jiao to cubilose is 2:3; E:C = 4:3, the mass ratio of E'jiao to cubilose is 4:3.

By evaluating the free radical scavenging abilities of E'jiao, cubilose, and their combination, it has been observed that the complex demonstrates a moderate ability to scavenge free radicals after simulating in vitro digestion. However, there was no significant enhancement in the free radical clearance ability compared to the individual digestive fluids of E'jiao and cubilose. This finding suggests that the complex may exert its functionality through an optimal pathway or mode of action.

### 3.2 Analysis of the Antioxidant Capacities of the Digestive Fluids of E'jiao, Cubilose, and Their Combination

#### 3.2.1 Toxicity Analysis of the Digestive Fluids of E'jiao, Cubilose, and Their Combination on HSF Cells

**FIGURE 1:**
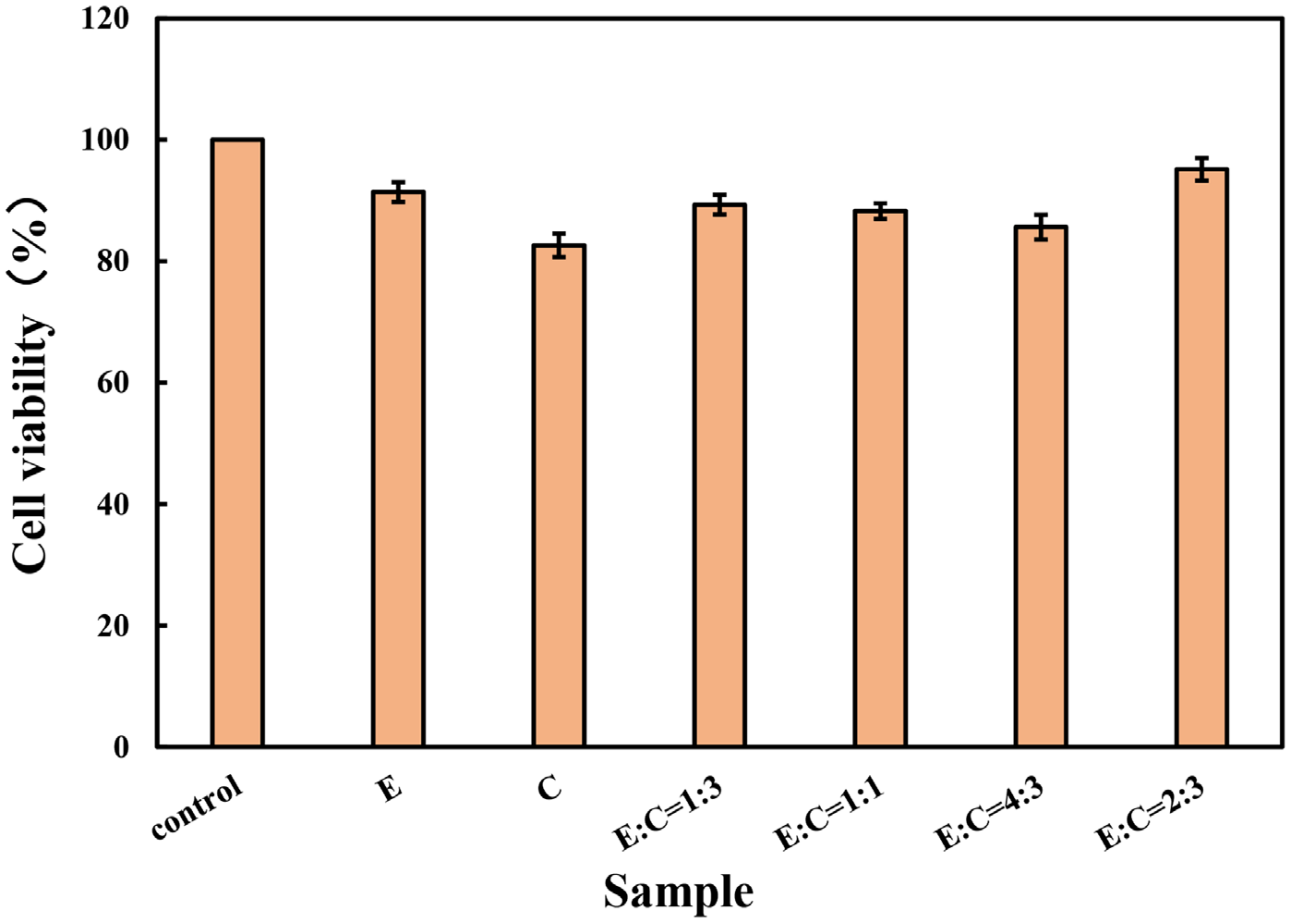
The impact of different samples on the cell viability of HSF cells. C, cubilose; E, E'jiao; E:C = 1:3, the mass ratio of E'jiao to cubilose is 1:3; E:C = 1:3, the mass ratio of E'jiao to cubilose is 1:1; E:C = 4:3, the mass ratio of E'jiao to cubilose is 4:3; E:C = 2:3, the mass ratio of E'jiao to cubilose is 2:3. Values are presented as mean ± SD (*n* = 3).

To evaluate the impact of the digestive fluids of E'jiao, cubilose, and their combination on the viability of HSF cells, cell viability was measured. The results, as shown in Figure 1, indicated that the digestive fluids of all tested samples can maintain HSF cell survival rate above 80%. This suggests that these samples' digestive fluids exhibit low cytotoxicity and can be used for further cellular research.

#### 3.2.2 Screening of H_2_O_2_ Concentrations in the H_2_O_2_ ‐HSF Cell Model

**FIGURE 2:**
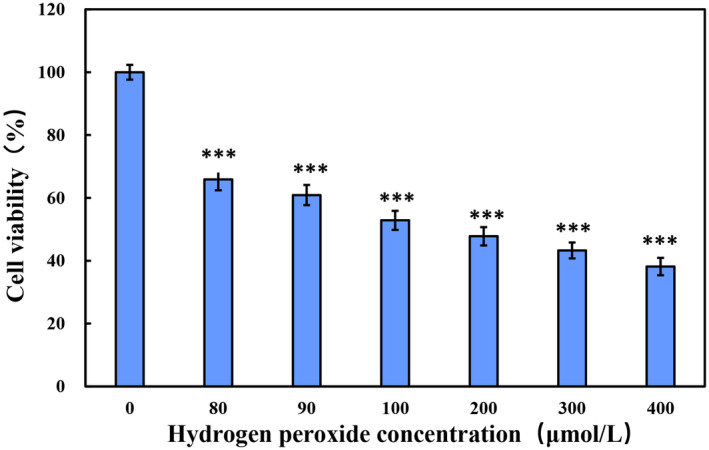
The effect of different concentrations of hydrogen peroxide on the cell viability of HSF cells. C, cubilose; E: E'jiao; E:C = 1:3, the mass ratio of E'jiao to cubilose is 1:3; E:C = 1:3, the mass ratio of E'jiao to cubilose is 1:1; E:C = 4:3, the mass ratio of E'jiao to cubilose is 4:3; E:C = 2:3, the mass ratio of E'jiao to cubilose is 2:3. Values are presented as mean ± SD (*n* = 3). *Significance analysis of different concentrations of hydrogen peroxide treatment groups compared to the control group: ***p < 0.001.

To further explore the mechanisms by which E'jiao, cubilose, and their combination defend against oxidative damage, H_2_O_2_‐treated HSF cells were used to establish an oxidative damage model. As shown in Figure 2, treating HSF cells with different concentrations of H_2_O_2_ (0, 80, 100, 200, 300, and 400 μmol/L) after 1 h, cell viability significantly decreased compared to the control group (no H_2_O_2_ added) (*p* < 0.001) and exhibited a concentration‐dependent effect. When the final concentration of hydrogen peroxide was 100 μmol/L, the cell viability of HSF cells was 52.86%. In subsequent studies, 100 μmol/L hydrogen peroxide was used as the inducing concentration.

#### 3.2.3 Effects of Digestive Fluids of E'jiao, Cubilose, and Their Combination on the Activities of Glutathione Peroxidase, Catalase, and Superoxide Dismutase in H_2_O_2_ ‐Treated HSF Cells

When the body experiences mild oxidative stress, its inherent antioxidant defense system is activated to eliminate free radicals and protect against damage from external factors. This response helps to maintain the body's redox balance [24]. However, when the effects of external stimuli exceed the body's intrinsic regulatory capacity, severe oxidative damage can occur [25]. The cellular antioxidant defense system primarily relies on the collective action of various antioxidant enzymes (SOD, GSH‐PX, and CAT) to counteract oxidative stress. Therefore, in this study, the protective effects of different sample groups on oxidative damage in cells were evaluated by measuring changes in antioxidant enzyme activity.

**FIGURE 3:**
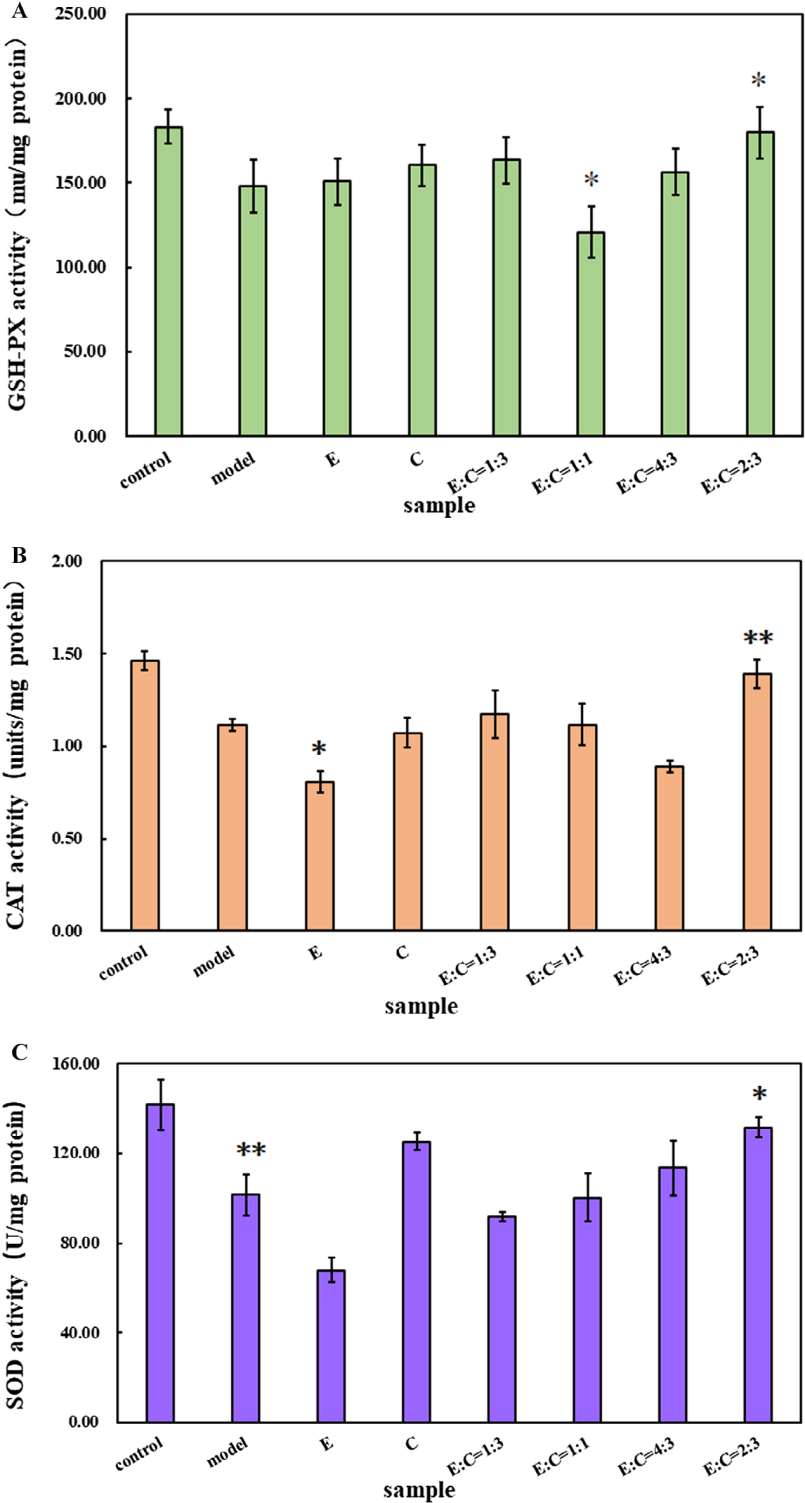
Effects of different samples on GSH‐PX, CAT, and SOD enzyme activities in H_2_O_2_‐induced HSF cells. (A), GSH‐PX(B), CAT(C), and SOD. Control, blank control group; Model, model group; E, E'jiao; (C), cubilose; E:C = 1:3, the mass ratio of E'jiao to cubilose is 1:3; E:C = 1:3, the mass ratio of E'jiao to cubilose is 1:1; E:C = 4:3, the mass ratio of E'jiao to cubilose is 4:3; E:C = 2:3, The mass ratio of E'jiao to cubilose is 2:3. Values are presented as mean ± SD (*n* = 3). *Significance analysis of different concentrations of hydrogen peroxide treatment groups compared to the model group: *p < 0.05, **p < 0.01.

The digestive fluid antioxidant enzyme activities of E'jiao, cubilose, and their combination are shown in Figure 3. It can be seen that after H_2_O_2_ induction, intracellular antioxidant enzyme activities were lower than the control group, indicating successful model construction. When E'jiao and cubilose were combined in a ratio of 1:1, the protective effect on glutathione peroxidase was poor, with an enzyme activity of 120.69 mU/mg, significantly lower than the model group (*p* < 0.05). When the combination ratio of E'jiao and cubilose was 1:3 and 4:3, the combined product showed good improvement in the activities of all three enzymes. When the combination ratio was 2:3, the combined product exhibited significantly increased SOD, GSH‐PX, and CAT enzyme activities compared to the model group (*p* < 0.01, *p* < 0.05), reaching 131.51, 179.88, and 1.39 U/mg, respectively. This indicates that the synergistic effect of E'jiao and cubilose is best under this combination condition, providing better protection against H_2_O_2_‐induced damage to the antioxidant defense system.

#### 3.2.4 Effects of Digestate of E'jiao, Cubilose, and Their Combination on ROS Levels and Cell Membrane Permeability in H_2_O_2_ ‐Induced HSF Cells

**FIGURE 4:**
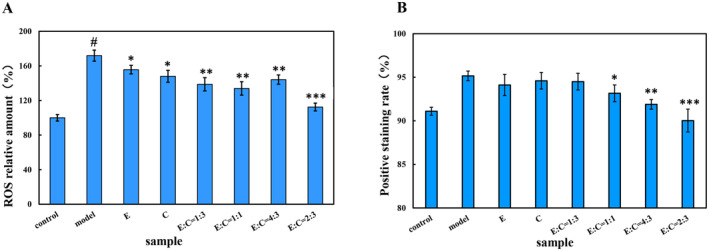
The effects of different samples on ROS levels and cell membrane permeability in H_2_O_2_‐induced HSF cells were evaluated (A). The impact on ROS levels in HSF cells (B). The effects of different samples on cell membrane permeability in H_2_O_2_‐induced HSF cells. Control, blank control group; Model, model group; E, E'jiao; C, cubilose; E:C = 1:3, the mass ratio of E'jiao to cubilose is 1:3; E:C = 1:3, the mass ratio of E'jiao to cubilose is 1:1; E:C = 4:3, the mass ratio of E'jiao to cubilose is 4:3; E:C = 2:3, the mass ratio of E'jiao to cubilose is 2:3. Values are presented as mean ± SD(*n* = 3). **#**Statistical analysis comparing the significance between the experimental group and the blank control group. *Significance analysis of different concentrations of hydrogen peroxide treatment groups compared to the model group: *p < 0.05, **p < 0.01, and ***p < 0.001.

To investigate the relationship between the protective effects of E'jiao, cubilose, and their combination on HSF cell damage and the reduction of oxidative stress, intracellular ROS levels were measured using the DCFH‐DA fluorescent probe. The results, shown in Figure 4A, indicated a significant increase in ROS secretion in the model group (*p* < 0.05), reaching 171.87% compared to the blank control group (100%). This suggests that treatment of cells with H_2_O_2_ solution leads to an elevation of ROS levels in HSF cells. However, when the digestate of E'jiao and cubilose were applied to H_2_O_2_‐induced HSF cells, ROS levels significantly decreased compared to the model group (*p* < 0.05). After treatment with different combinations 1:3, 1:1, and 4:3, there was a significant decrease in ROS levels compared to the model group (*p* < 0.01). Cells treated with the digestate in a 2:3 combination showed a significant reduction in ROS levels compared to the model group (*p* < 0.001), reaching 112.42%. This indicates that the digestate of E'jiao, cubilose, and their combination can directly eliminate intracellular ROS to alleviate the degree of cellular oxidative damage, with the best ROS elimination effect observed when the combination ratio is 2:3.

To study the protective effects of E'jiao, cubilose, and their combination on cell membrane integrity, we analyzed their impact on cell membrane permeability in HSF cells. The permeability of cell membranes is influenced by an excessive amount of ROS and can have implications for cellular apoptosis. The results shown in Figure 4B revealed that the blank control group had the lowest staining rate at 91.10%, while the model group exhibited the highest staining rate at 171.87%, indicating that oxidative damage enhances cell membrane permeability. After treatment with E'jiao and cubilose digestate, the staining rate significantly decreased compared to the model group (*p* < 0.05). Cells treated with the digestate of different combinations 1:3, 1:1, and 4:3 also showed a significant reduction in staining rate compared to the model group (*p* < 0.01). Particularly, cells treated with the digestate in a 2:3 combination exhibited a staining rate of 112.42%, similar to the blank control group and significantly lower than the model group (*p* < 0.001). This indicates that the digestate of E'jiao, cubilose, and their combination can effectively mitigate the impact of oxidative damage on cell membrane integrity, with the 2:3 combination ratio showing the most efficient protection of HSF cell integrity.

In summary, H_2_O_2_ can disrupt cell membrane integrity, accelerate lipid peroxidation processes, and inhibit the activity of intracellular antioxidant enzymes CAT, SOD, and GSH‐PX. However, treatment with different ratios of digestive fluid can inhibit lipid peroxidation processes, maintain cell membrane integrity, and significantly enhance antioxidant enzyme activity. When the combination ratio of E'jiao and cubilose was 2:3, the combination product showed better ability to resist damage caused by H_2_O_2_ to the antioxidant defense system compared to other groups of digestive fluid.

### 3.3 Effect of the Combination Product of E'jiao and Cubilose (at a Ratio of 2:3) on the Cell Cycle of H_2_O_2_ ‐Induced HSF Cells

**FIGURE 5:**
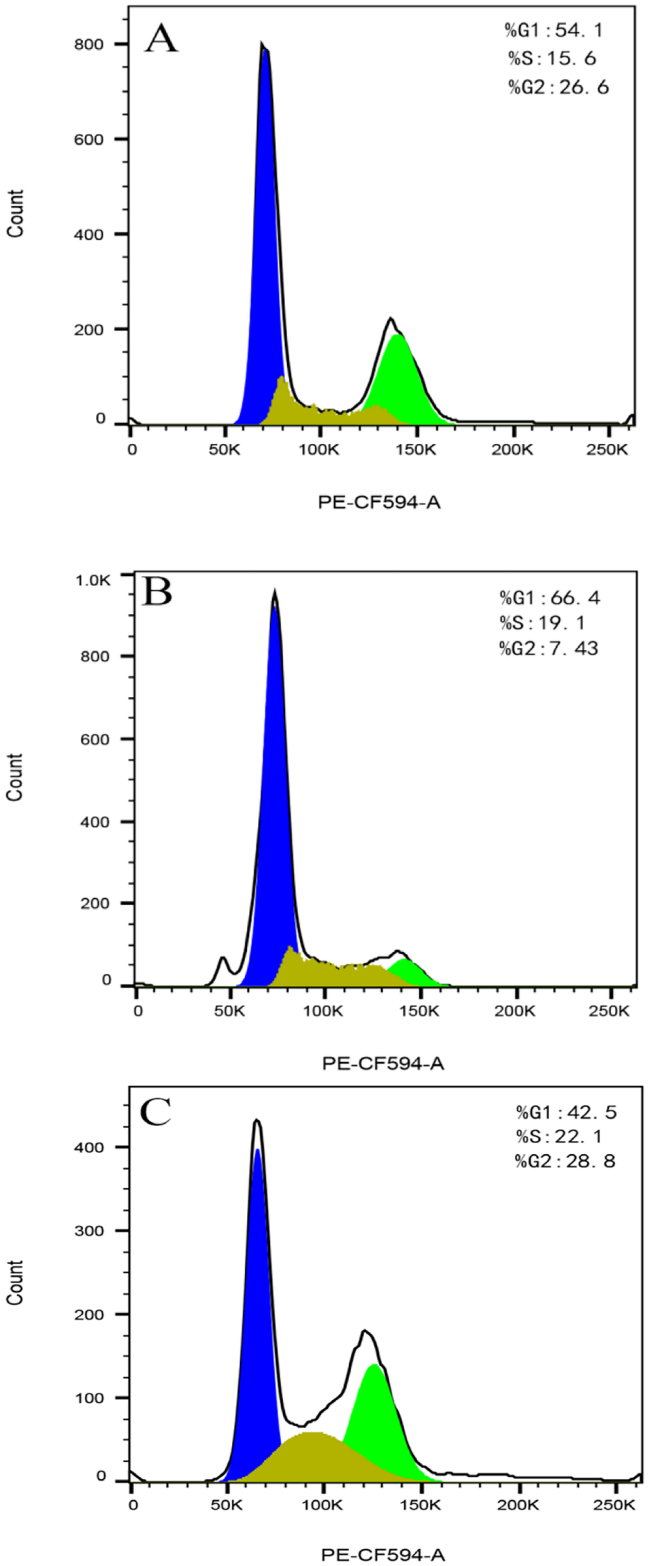
Cell cycle diagram of different treatment groups (A) control group; (B) model group: H_2_O_2_ treatment for 1 h; and (C) compound treatment group: After adding the compound of E'jiao and cubilose, the digestive fluid mixed with a mass ratio of 2:3 was treated for 24 h, and H_2_O_2_ was added for 1 h.

In the previous comprehensive evaluation of the antioxidant capacities of E'jiao, cubilose, and their combination, the sample with a combination ratio of 2:3 demonstrated excellent performance. To further elucidate the mechanism of action of this combination product on cellular oxidative stress, its effect on the cell cycle of H_2_O_2_‐induced HSF cells is shown in Figure 5. The first peak in the graph represents the G0/G1 phase, the middle trough represents the S phase, and the second peak represents the G2/M phase. As shown in Figure 5B, the percentage of cells in the S%+G2/M% phase after 1 h of H_2_O_2_ treatment was 19.1% + 7.4%, lower than the blank group (15.6% + 26.6%) (Figure 5A). This indicated that H_2_O_2_ treatment arrested HSF cells at the G0/G1 phase. However, after the addition of the combination product digestive fluid with a ratio of 2:3, the percentage of cells in the S%+G2/M% phase increased to 22.1% + 28.8%, higher than the model group (19.1% + 7.4%), suggesting that this combination product digestive fluid can help damaged cells recover their proliferative capacity to some extent.

### 3.4 Effects of the Combination Product of E'jiao and Cubilose (Mass Ratio of 2:3) on Collagen I and Collagen IV in a 3D Full‐Skin Model

In the previous evaluations of the antioxidant capacities of E'jiao and cubilose combination products, as well as its ability to protect HSF cells from oxidative damage, it was found that the combination product at a ratio of 2:3 exhibited more comprehensive and superior antioxidant abilities. To better understand the antiwrinkle effects of this combination product on the living full‐skin layer, a more comprehensive efficacy assessment was conducted using a three‐dimensional skin equivalents model.

#### 3.4.1 The Results of Cell Toxicity

**FIGURE 6:**
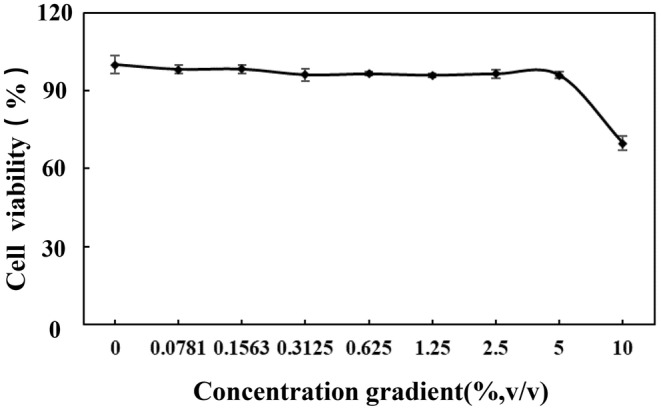
The impact of the digestive solution of the combination product at a ratio of 2:3 on the viability of fibroblast cells.

From the data in Figure 6, it can be observed that the working concentration of the combination product should be equal to or < 5% for safety reasons. A working concentration of 5% (v/v) was chosen for evaluating the antiwrinkle efficacy on the full‐skin layer.

#### 3.4.2 The Impact of the Combination Product on the Collagen Type I Content in the 3D Full‐Skin Layer Treated With UVA

**FIGURE 7:**
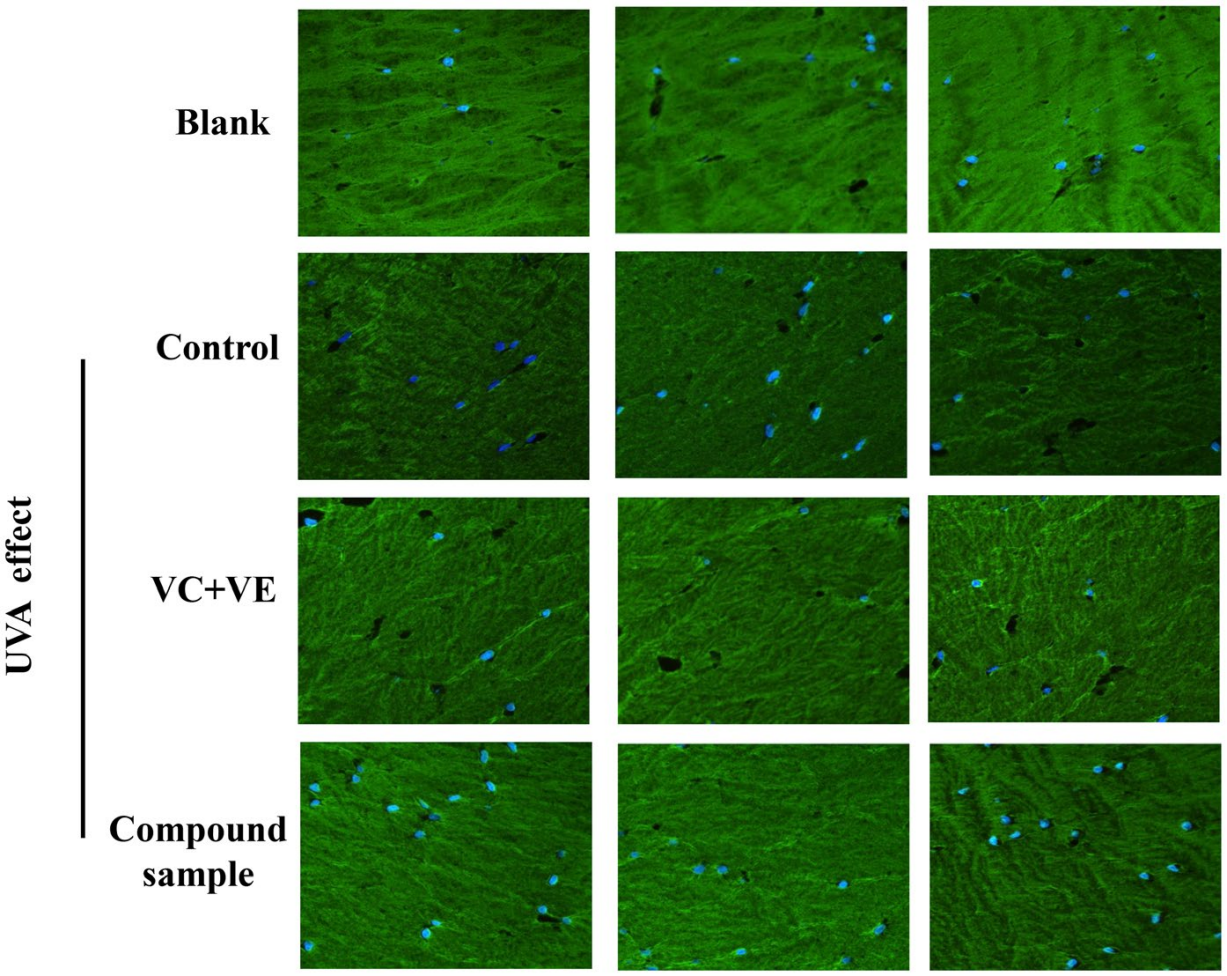
Immunofluorescence image of collagen Type I in the 3D full‐skin layer.

**FIGURE 8:**
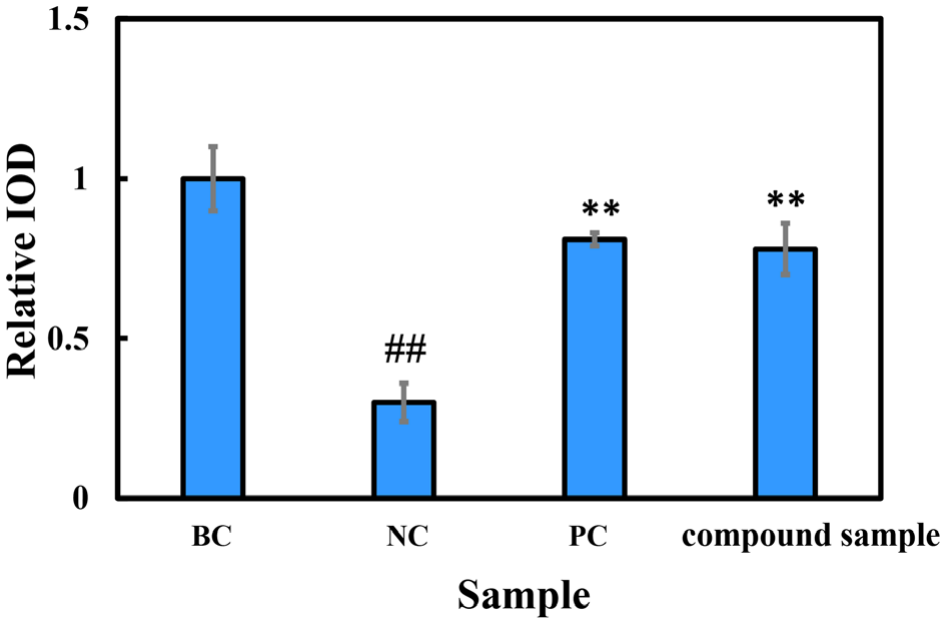
The effect of the combination product, a mixture of E'jiao and cubilose in a 2:3 mass ratio, on the collagen Type I content in the 3D full‐skin layer treated with UVA. BC, blank control; NC, negative control; PC, positive control; Compound sample: A mixture of E'jiao and cubilose in a 2:3 mass ratio. when comparing the NC group with the BC group: ^##^ *p* < 0.01. When comparing the PC group and the sample group with the NC group: **p < 0.01.

To further investigate the mechanism of action of the combination product of E'jiao and cubilose, we utilized immunofluorescence staining techniques to compare the expression of characteristic proteins in the skin using a 3D skin model. According to the immunofluorescence staining of collagen Type I in the 3D skin model (Figure 7), the fluorescence intensity was significantly higher in the group treated with the combination product compared to the negative control group without any treatment. Quantitative analysis of the fluorescence data showed a significant increase in the relative integrated optical density (IOD) value in the combination product group compared to the negative control group, with a 160% increase, which was similar to the positive control groups treated with vitamin C (VC) and vitamin E (VE) (Figure 8). These results indicate that the combination product has a promoting effect on the expression of collagen Type I.

#### 3.4.3 The Effect of the Combination Product on the Collagen Type IV Content in the 3D Full‐Skin Layer Treated With UVA

**FIGURE 9:**
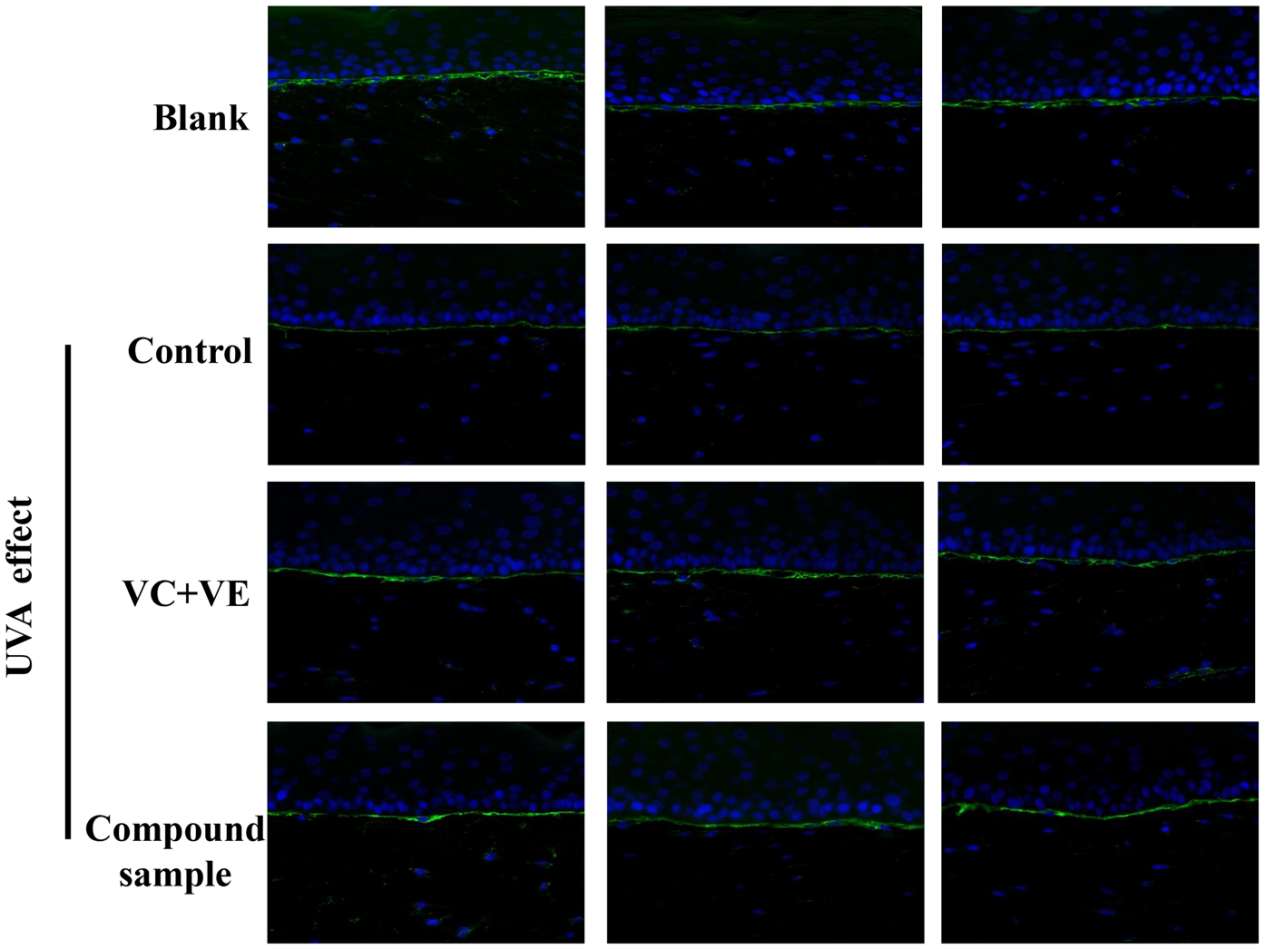
The immunofluorescence image of collagen Type IV in the 3D full‐skin layer.

**FIGURE 10:**
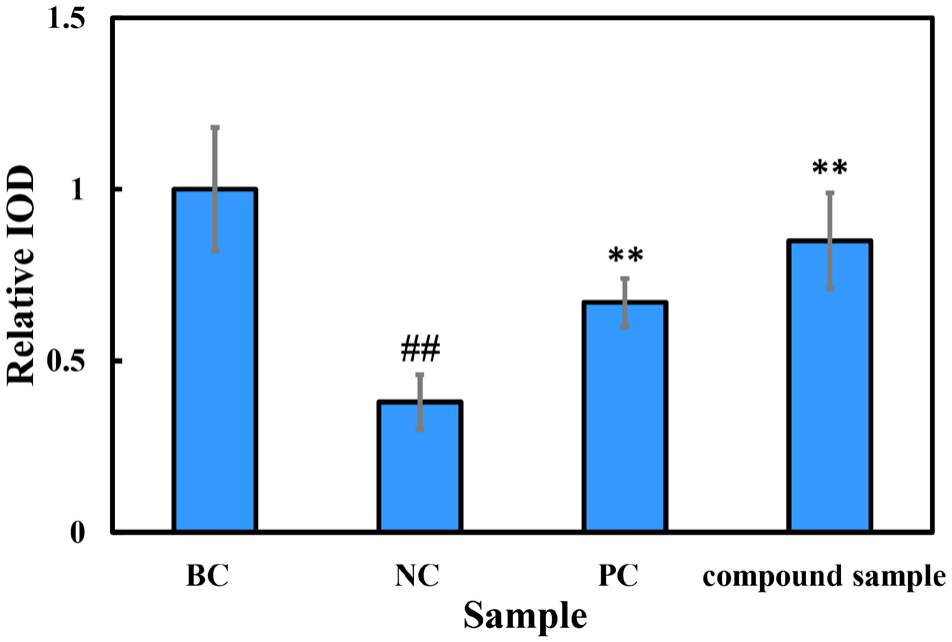
The effect of the combination product, a mixture of E'jiao and cubilose with a mass ratio of 2:3, on the collagen Type IV content in the 3D full‐skin layer treated with UVA. BC, blank control; NC, negative control; PC, positive control; Compound sample: A mixture of E'jiao and cubilose in a 2:3 mass ratio. When comparing the NC group with the BC group: ##*p* < 0.01. When comparing the PC group and the sample group with the NC group: **p < 0.01.

Collagen Type IV is an important component of the basement membrane, and its expression level partly reflects the health status of the dermis in the skin. Immunofluorescence staining of collagen Type IV in the 3D skin model treated with the combination product is shown in Figure 9. The sample group treated with the digestion solution containing the combination product showed a noticeable increase in the expression of collagen Type IV compared to the negative control group without the sample. The relative IOD value of the sample group with the combination product was significantly elevated, showing an increase of 123.68% compared to the negative control group (Figure 10).

The 3D skin model constructed in vitro in this study closely resembles normal human skin in structure. It can serve as a substitute for human skin in evaluating the expression of important proteins in response to active substances or other stimuli. Therefore, it can be used as an alternative to human skin for assessing the efficacy of cosmetic actives. Based on the above results, it can be concluded that the digestion solution with a combination ratio of 2:3 promotes the expression of collagen Type I and collagen Type IV, with an increase of 168.00% and 123.68%, respectively.

## 4 Discussion

E'jiao and cubilose are traditional Chinese health food products with various effects such as anti‐inflammatory, antioxidant, and skin health improvement. To obtain the optimal ratio of their compounds, this study comprehensively evaluated them from the perspectives of in vitro antioxidant capacity, cellular antioxidant damage capacity, and 3D whole skin modeling. It was found that the composite of E'jiao and cubilose with a compound ratio of 2:3 was not significant in terms of in vitro antioxidant, but it was significant (*p* < 0.05) when assessed in terms of antioxidant damage capacity using cells as a model. Furthermore, the complex significantly increased Type I collagen and Type IV collagen content in 3D skin modeling studies.

Free radicals are atoms, molecules, ions, or groups of atoms with unpaired electrons. Many external factors such as ultraviolet radiation, high temperature, and low oxygen can stimulate cells to produce a large number of free radicals, leading to oxidative damage in cells. Tang, Goh, and Sia showed that the unsaturated fatty acids in cubilose can enhance its antioxidant capacity by scavenging DPPH free radicals [26]. In a study conducted by Cao et al., it was found that peptides in cubilose can effectively scavenge DPPH free radicals and superoxide anion free radicals [27]. According to the research conducted by Ogasawara et al., it was shown that sialic acid in cubilose exhibits antioxidant effects by scavenging hydroxyl radicals [28]. In this study, the digests of E'jiao, cubilose alone, and its complexes had the ability to scavenge DPPH radicals, superoxide anions, and hydroxyl radicals (Table 1), which was consistent with the literature reports. However, we also found that the free radical scavenging ability of the digestate of the compound was not significantly enhanced or slightly decreased compared with the single product. This may be related to the complexity of the mechanism of action between antioxidants and free radicals or the fact that the digests of the complexes do not reflect their antioxidant functional advantages well in the in vitro model, and they need to enter the cells and be metabolized in vivo to reflect their antioxidant capacity better.

Digestive compounds from food or drugs are typically absorbed into the bloodstream and then distributed to tissues and cells. According to the research conducted by Hou et al., it was found that cubilose can enhance the activities of superoxide dismutase (SOD) and increase SOD‐1 mRNA levels in SH‐SY5Y cells under oxidative stress induced by H_2_O_2_ [29]. Gupta et al. found that cubilose can reduce the production of ROS in HaCat and SH‐SY5Y cells [30]. Other studies have indicated that consuming E'jiao can restore the antioxidant defense system by increasing the activity of antioxidant enzymes [6]. In this study, we further evaluated the antioxidant capacity of E'jiao, cubilose, and their complexes in vivo using H_2_O_2_‐induced HSF cells as a model of oxidative damage. It was found that when the compound ratio of E'jiao to cubilose was 2:3, it could significantly inhibit the lipid peroxidation process, maintain the integrity of cell membranes, enhance the activities of antioxidant enzymes, and change the cell cycle changes induced by H_2_O_2_, which was the optimal ratio under the in vivo model. Under the condition of this compounding ratio, the bioactive substances of the whole digestive system can be better superimposed, stimulate the expression of oxidative enzymes at the mRNA level, and reduce the content of ROS in the cells, which contributes to the antioxidant capacity of this complex digestive solution being higher than the rest of the samples.

3D skin modeling is the application of cell biology and engineering techniques and principles, the use of normal human skin cells in vitro to obtain a complete three‐dimensional anatomical structure, and a high degree of simulation of the human skin tissue models [31]. Nowadays, 3D skin modeling has not only been used in clinical treatment of burns but also in the evaluation of the efficacy of cosmetic raw materials and finished products [32]. Type I collagen the most abundant collagen in the skin provides tensile strength and stability to the dermis and plays an important role in the elasticity of the skin. Type IV collagen forms a honeycomb hexagonal structure mainly in the basement membrane, which plays an important role in the prevention of wrinkle formation. According to reports, cubilose and digestive fluid can attenuate the activation of the JNK/c‐FOS/c‐Jun/MMP pathway under UVB irradiation, while stimulating the activation of the TGF‐βRI/SMAD3/procollagen Type I pathway [8]; cubilose digest also regulates mitogen‐activated protein kinase and activator protein‐1 pathways to inhibit the expression of MMPs, maintaining skin elasticity and slowing down the aging process [33]. In addition, E'jiao prevented H_2_O_2_‐induced delayed wound healing, reduced UVA‐decreased Type IV collagen and elastin synthesis, and reduced UVA‐induced wrinkle formation [7]. In this study, we found that a combination of E'jiao and cubilose extract at a ratio of 2:3 in a 3D skin model increased the levels of Type I and Type IV collagen, consistent with previous literature reports. The combined formulation showed increased levels of total amino acids, polysaccharides, and proteins compared to individual components. Additionally, the spatial arrangement of the components in this combination ratio was more reasonable, enhancing the synergistic effect of E'jiao and cubilose extract. Under the influence of this combination, the synthesis pathways of Type I and Type IV collagen were simultaneously activated, resulting in increased expression levels. This strengthened the integrity of the dermal–epidermal junction (DEJ) and influenced substance exchange among the DEJ, epidermis, and dermis, ultimately improving the activity and regenerative capacity of the epidermal cells. The combination formulation exhibited antiwrinkle effects.

This study comprehensively utilized in vitro antioxidant assays, H_2_O_2_‐HSF cell model, and 3D full‐skin model to demonstrate that the digestive fluid of the E'jiao and cubilose complex participates in in vivo metabolism in the form of small molecules. Activation of relevant metabolic pathways is the main mechanism underlying its antioxidant effects. It was determined that the optimal ratio of E'jiao to cubilose is 2:3 in terms of antioxidant capacity. It provides new insights and perspectives for the application of E'jiao and cubilose complex.

## Author Contributions

**Hang Tie** and **Xiao Xie:** conceptualization. **Zichen Zhang:** methodology. **Jianling Zhang:** validation. **Zichen Zhang:** data curation. **Hang Tie** and **Xiao Xie:** writing – original draft preparation. **Liang Xu** and **Haihua Ruan** writing – review and editing. **Hongyang Zhang** and **Tao Wu:** supervision. All authors have read and agreed to the published version of the manuscript.

## Conflicts of Interest

The authors declare no conflicts of interest.

## Data Availability

The data that support the findings of this study are available from the corresponding author upon reasonable request.
